# Ten-year survival rate after rotational acetabular osteotomy in adulthood hip dysplasia

**DOI:** 10.1186/s12891-017-1556-7

**Published:** 2017-05-15

**Authors:** Masamitsu Tomioka, Yutaka Inaba, Naomi Kobayashi, Taro Tezuka, Hyonmin Choe, Hiroyuki Ike, Tomoyuki Saito

**Affiliations:** Department of Orthopedic Surgery, Yokohama City University, 3-9 Fukuura, Kanazawa-ku, Yokohama, Japan

**Keywords:** Rotational acetabular osteotomy, Adulthood hip dysplasia, Osteoarthritis, Survival analysis, Risk factors

## Abstract

**Background:**

Rotational acetabular osteotomy (RAO) is an effective joint-preserving surgical treatment for adulthood hip dysplasia (AHD). Despite sufficient correction of acetabular dysplasia, some patients still experience osteoarthritis (OA) progression and require total hip arthroplasty (THA). The purposes of the current study were to investigate the survival rate and the risk factors for OA progression or THA requirement after RAO and to explore whether acetabular overcorrection relates to OA progression.

**Methods:**

Fifty-six patients (65 hips, mean age: 36.5 ± 11.7 years) with AHD who underwent RAO and were followed up for >10 years (mean: 15.0 ± 3.2 years) were enrolled in this study. A Kaplan-Meier survival analysis was performed to assess the non-OA progression rate and THA-free survival rate of RAO during the 10-year follow-up. To analyze the risk factors for OA progression and THA requirement, the Cox proportional hazards regression analysis was performed.

**Results:**

No OA progression was found in 76.7% of the patients, and THA was not required in 92.3% during the 10-year follow-up. By multivariate regression analysis, older age at the time of surgery was a risk factor for both OA progression (hazard ratio [HR] = 1.047, 95% confidence interval [CI] = 1.005–1.091) and THA requirement (HR = 1.293, 95% CI = 1.041–1.606).

**Conclusion:**

RAO is an effective surgical procedure for symptomatic patients with AHD that prevents OA progression and protects the hips from undergoing THA. However, older patients have a higher risk for both OA progression and THA requirement.

## Background

Adulthood hip dysplasia (AHD) is a hip condition that causes hip pain and osteoarthritis (OA). AHD is one of the major causes of hip OA in the Japanese population [1]. Dysplastic acetabular roof induces instability and incongruence of the hip joints at an early age that trigger the progression of hip OA and may require total hip arthroplasty (THA) even in young- or middle-aged patients. Since THA for young- or middle-aged active patients is associated with high rates of loosening [2, 3], joint-preserving operations are an important surgical option.

Rotational acetabular osteotomy (RAO) is a joint-preserving periacetabular osteotomy technique [4–8] developed by Ninomiya and Tagawa [9] that corrects insufficient femoral coverage by rotating the acetabular roof with an articular cartilage. RAO remains a successful treatment for patients with AHD with careful selection of indications [10]. However, some reported that older age at the time of surgery is associated with lower success rates in this procedure [11]. Since many factors affect the outcome of RAO, the optimal indication is still controversial, especially in middle-aged patients. Appropriate surgical technique and patient selection are important factors for successful outcomes. Undercorrection of the acetabular roof may result in instabilities of the hip joint, and efficient coverage of the acetabular roof to the femoral head is essential in this procedure. However, overcorrection of the acetabular roof may lead to pincer impingement and accelerate OA progression. Association of overcorrection and OA progression after RAO was rarely investigated previously [6, 12].

The purposes of the current study were to investigate the 10-year survival rate and risk factors for OA progression or THA requirement after RAO and whether overcorrection of the acetabular roof accelerates OA progression in our patients.

## Methods

The study was approved by the authors’ institutional review board (Yokohama City University, #B170100004), and informed consent for participating the current study and for publication was obtained from all patients. Fifty-six patients (4 men, 52 women; 65 hips) who underwent RAO at our institution and were followed up for > 10 years were enrolled in the current study. Indications of the surgery were as follows: 1. age < 60 years, 2. progressive pain that interferes with daily activities, 3. center edge (CE angle) < 20°, and 4. improvement of the femoral head coverage and joint congruity in a hip abduction position on anteroposterior hip radiography. One patient (1 hip) had a neurological disorder due to cerebral palsy as a comorbidity. In these 56 patients, preoperative and postoperative radiographs, medical records, and operative reports were reviewed retrospectively. In cases with deformities of the femoral head, we performed intertrochanteric femoral varus or valgus osteotomy combined with RAO to improve the joint congruency. In the current study, 11 hips of 10 patients had a simultaneous intertrochanteric osteotomy. One had a varus osteotomy, and the other 10 hips had a valgus osteotomy.

As an operative technique and rehabilitation, a 15 to 20 cm straight incision was made from the distal aspect of the greater trochanter toward the iliac crest. In order to approach the superior aspect of the acetabulum, a transtrochanteric approach was performed. The osteotomy line of the acetabulum was marked at about 1.5 cm from the acetabular rim, and an osteotome was inserted to a depth of about 1.0 cm without penetrating the joint. A curved osteotome was then used to avoid penetrating the inner wall of the ilium, and the osteotomy was performed around the circumference of the acetabulum. Adequate coverage of the femoral head was achieved by moving the resected acetabulum laterally and anteriorly to make a 0° acetabular roof obliquity (AC angle). Two or 3 absorbable screws were used to fix the rotated acetabulum to the ilium. The greater trochanter was reattached using 2 absorbable screws; one was 6.5 mm and the other was 4.5 mm in diameter, and the screws were fixed bicortically. In cases of an additional trochanteric valgus osteotomy, we exposed the proximal shaft of the femur and inserted the screw toward the femoral head at the predetermined insertion angle under image intensification. Thereafter, lateral wedge osteotomy was performed at the level of the lesser trochanter. After removal of the lateral wedge and subsequent valgization, the removed wedge bone was implanted between the femur and resected greater trochanter to restore the femoral offset. The distal femur was then fixed with a plate and the greater trochanter with 2 pairs of pins and cables.

Transfer in a wheelchair was begun 1 week after surgery. One-third weight-bearing positions were allowed at 3 weeks, half weight-bearing at 5 weeks, and full weight walking at 8 weeks after surgery.

Need for THA because of a painful hip after RAO was defined as the clinical end point of the follow-up. The OA stage was radiographically divided into 4 stages, according to the classification of the Japanese Orthopedic Association (JOA) (Prearthritis: acetabular dysplasia without osteoarthritic changes; Early stage: slight narrowing of the joint space associated with sclerosis of the subchondral bone; Advanced stage: narrowing of the joint space with cystic lucencies and small osteophytes; and End stage: disappearance of the joint space and marked osteophyte formation) [4]. The diagnosis of OA progression was made by comparing the OA stage before surgery and at the final follow-up.

Anteroposterior hip radiographic assessments in the supine position were conducted. A single well-trained orthopedic surgeon, who was blinded from the clinical results, performed all radiographic measurements. For the assessment of acetabular coverage on the femoral head, CE angle, sharp angle, acetabular femoral head index, and AC angle were quantified. Joint space width (JSW), presence of cysts, deformity of the femoral head, and thickness of the transferred acetabulum at the widest portion were also measured. Obturator foramen-head distance, which is the distance from the lateral side of the obturator foramen to the medial side of the femoral head that represents an index of medialization of the hip, was also quantified. The abovementioned radiographic assessments were conducted before and immediately after surgery. Radiographic assessment of acetabular coverage was also performed at the final follow-up.

We also assigned our patients to two groups: the overcorrection and non-overcorrection groups, according to the anteroposterior hip radiographic measurement using the CE angle immediately after RAO [13]; we defined overcorrection as a CE angle > 40° and non-overcorrection as a CE angle ≤ 40° according to the reports of Chung et al. [14] Survival rates for OA progression and THA requirement in the overcorrection and non-overcorrection groups were compared.

We also evaluated the influence of smoking on the result of RAO. Patients were also divided into non-smokers and smokers, according to the Center for Disease Control and Prevention guidelines to assess the risk of nicotine abuse [15].

A Kaplan-Meier survival analysis was performed to assess the non-OA progression rate and THA-free survival rate of RAO during the 10-year follow-up. To analyze the risk factors for OA progression and THA requirement, the Cox proportional hazards regression analysis was performed. Cox regression analysis was firstly performed univariately on potential risk factors. Furthermore, risk factors that indicate significant *p* values < 0.05 were populated for the Cox regression analysis as a multivariate assessment. We calculated the cut off points of each variable which indicated significant *p* value < 0.05 for both THA requirement and OA progression with the receiver operating characteristic (ROC) curve. To analyze the time course difference of mean normal variance data, the paired *t*-test was performed. To analyze the influence of an additional intertrochanteric valgus or varus osteotomy, we performed a log-rank analysis and compared the non-OA progression rate and THA-free survival rate of RAO. A *p* value < 0.05 was considered statistically significant. All analyses were performed using the SPSS version 22.0 (IBM, Armonk, NY, USA).

## Results

The mean age at the time of surgery was 36.5 ± 11.7 years (range, 11–56 years). The mean body mass index was 21.2 ± 2.3 kg/m^2^ (range, 16.9–33.5 kg/m^2^). Twelve patients (21.4%) were smokers. Ten patients (11 hips) were treated with intertrochanteric valgus or varus osteotomy simultaneous to the RAO procedure to improve joint congruency. The average follow-up period was 15 ± 3.2 years (range, 10–22.5 years). In all hips, coverage of the acetabulum on the femoral head improved after surgery (Table 1).

**Table 1:** Radiological measurements of acetabular coverage of the femoral head before and after surgery

	Preoperative	Immediately after surgery	*p* value (paired *t*-test)
Center edge angle (°)	6.0 ± 10.7	49.9 ± 13.1	<0.001
Sharp angle (°)	48.9 ± 4.1	32.5 ± 7.7	<0.001
Acetabular head index (%)	56.8 ± 10.1	97.9 ± 10.8	0.001
Acetabular roof obliquity (°)	27.2 ± 6.8	0.0 ± 12.1	<0.001
Obturator foramen-head distance (mm)	17.5 ± 3.5	15.6 ± 4.6	0.006

*N* = 65 hips

**Fig. 1:**
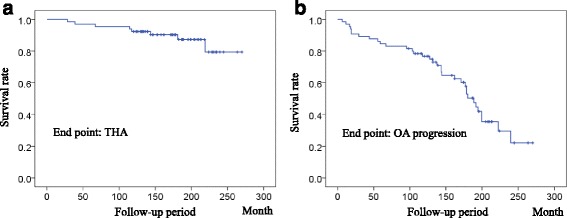
Kaplan-Meier survival analysis of rotational acetabular osteotomy for total hip arthroplasty (THA) requirement and osteoarthritis (OA) progression. During the 10-year follow-up, 92.3% of the hips did not need THA (**a**), and OA progression was not seen in 76.7% of the hips after RAO (**b**) (numbers at risk = 65 hips)

**Fig. 2:**
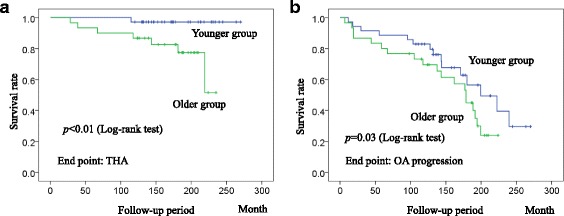
Kaplan-Meier survival analysis of rotational acetabular osteotomy for total hip arthroplasty (THA) requirement and osteoarthritis (OA) progression in the patients older than 41 years (older age group) and younger than 41 years (younger age group). The younger age group had significantly better THA-free survival rate (**a**) and OA progression rate (**b**) than the older age group during the 10-year follow-up (numbers at risk = 65 hips in the younger and older age groups) (Log-rank test, *p* < 0.01 and 0.03, respectively)

According to the JOA classification for OA grades, there were 46 Prearthritis, 15 Early, 4 Advanced, and no End stage cases, preoperatively. The 10-year unadjusted Kaplan-Meier survival rate of RAO for THA requirement was 92.3% (Fig. 1a). OA stage progressed in 15 hips during the 10-year follow-up, which included 5 THAs, and the 10-year survival rate for OA stage progression was 76.7% (Fig. 1b). To investigate the risk factors for OA progression or THA requirement, the Cox proportional hazards regression analysis was utilized. From the univariate Cox regression analysis of risk factors for THA requirement, age at the time of surgery, preoperative JSW, and radiographic stage indicated p values < 0.05. The Cox multivariate regression analysis revealed that the age at the time of surgery was an independent risk factor for THA requirement (hazard ratio [HR] = 1.293, *p* = 0.020, 95% confidence interval [CI] = 1.041–1.606) (Table 2). For the univariate Cox regression analysis, age at the time of surgery and thickness of the transferred acetabulum were significant risk factors for OA progression. However, the Cox multivariate regression analysis showed only age at the time of surgery as the independent risk factor for OA progression (HR = 1.047, *p* = 0.028, 95% CI = 1.005–1.091) (Table 3). We calculated the cut off points of age at the time of surgery for both THA requirement and OA progression with the ROC curve. The ROC curve showed the cut off points of 43.5 years for THA requirement (sensitivity: 87.5%; specificity: 66.7%) and 38.5 years for OA progression (sensitivity: 67.6%; specificity: 64.5%). Since age was found to be an important risk factor for poor outcomes in RAO, we divided all patients into 2 groups by age: >41 years (older age group) and ≤41 years (younger age group), according to the average of the cut off points calculated above. Seven hips required THA in the older age group during the 10-year follow-up and 1 hip in the younger age group. A log-rank analysis showed a significantly worse survival rate in the older age group for both OA progression and THA requirement (Fig. 2).

**Table 2:** Risk factors for THA requirement after RAO assessed by univariate and multivariate regression analyses

Variables	Hazard ratio	95% Confidence Interval	*p* value
Univariate regression			
Preoperative			
Age at the time of surgery	1.248	1.059–1.471	0.008
Body mass index	0.986	0.711–1.369	0.934
Smoking history	0.036	0.000–67.106	0.386
Presence of cysts	7.329	0.900–59.685	0.063
Deformity of the femoral head	0.515	0.063–4.208	0.536
Center edge angle (°)	0.958	0.909–1.011	0.119
Sharp angle (°)	1.056	0.892–1.250	0.527
Acetabular head index (%)	0.964	0.909–1.022	0.220
Acetabular roof obliquity (°)	1.023	0.923–1.134	0.662
Obturator foramen-head distance (mm)	1.200	0.972–1.481	0.091
Joint space width (mm)	0.655	0.449–0.957	0.029
Radiographic stage	2.691	1.205–6.013	0.016
Immediately after the surgery			
Center edge angle (°)	0.996	0.944–1.052	0.896
Sharp angle (°)	1.058	0.969–1.154	0.206
Acetabular head index (%)	0.992	0.931–1.057	0.803
Acetabular roof obliquity (°)	1.023	0.966–1.083	0.435
Obturator foramen-head distance (mm)	0.982	0.838–1.152	0.828
Joint space width (mm)	0.674	0.428–1.060	0.088
Radiographic stage	1.693	0.664–4.317	0.271
Joint congruity	1.981	0.898–4.372	0.091
Thickness of the transferred acetabulum	0.991	0.848–1.159	0.911
Multivariate regression			
Age at the time of surgery	1.293	1.041–1.606	0.020
Preoperative Joint space width	1.327	0.533–3.301	0.543
Radiographic stage	4.155	0.413–41.812	0.227

*N* = 65 hips

*THA* total hip arthroplasty, *RAO* rotational acetabular osteotomy

**Table 3:** Risk factors for OA progression after RAO assessed by univariate and multivariate regression analyses

Variables	Hazard ratio	95% Confidence Interval	*p* value
Univariate regression			
Preoperative			
Age at the time of surgery	1.051	1.011–1.093	0.013
Body mass index	0.996	0.847–1.170	0.957
Smoking history	0.555	0.213–1.451	0.230
Presence of cysts	1.398	0.710–2.752	0.333
Deformity of the femoral head	0.895	0.389–2.063	0.795
Center edge angle (°)	0.998	0.962–1.035	0.911
Sharp angle (°)	0.961	0.888–1.039	0.315
Acetabular head index (%)	1.000	0.963–1.037	0.981
Acetabular roof obliquity (°)	1.015	0.965–1.067	0.572
Obturator foramen-head distance (mm)	1.074	0.970–1.188	0.169
Joint space width (mm)	0.961	0.819–1.126	0.621
Radiographic stage	1.028	0.664–1.592	0.902
Immediately after the surgery			
Center edge angle (°)	0.996	0.971–1.023	0.783
Sharp angle (°)	1.014	0.972–1.058	0.520
Acetabular head index (%)	1.001	0.969–1.035	0.935
Acetabular roof obliquity (°)	0.999	0.974–1.026	0.959
Obturator foramen-head distance (mm)	0.925	0.855–1.001	0.052
Joint space width (mm)	0.953	0.763–1.189	0.669
Radiographic stage	0.808	0.459–1.425	0.462
Joint congruity	0.997	0.680–1.460	0.986
Thickness of the transferred acetabulum	0.887	0.800–0.982	0.022
Multivariate regression			
Age at the time of surgery	1.047	1.005–1.091	0.028
Thickness of the transferred acetabulum	0.923	0.829–1.027	0.142

*N* = 65 hips

*OA* osteoarthritis, *RAO* rotational acetabular osteotomy

**Fig. 3:**
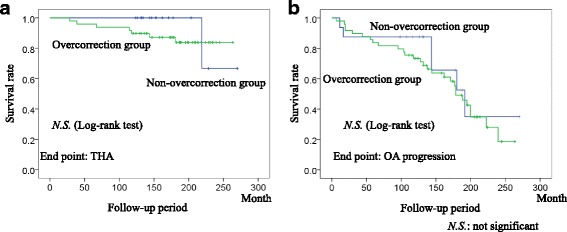
Kaplan-Meier survival analysis of rotational acetabular osteotomy for total hip arthroplasty (THA) requirement and osteoarthritis (OA) progression in the patients with postoperative center edge (CE) angle ≤ 40° (non-overcorrection group) and > 40° (overcorrection group). There was no significant difference in the THA-free survival rate between the 2 groups (**a**) and in the OA progression survival rate (**b**) during the 10-year follow-up (numbers at risk = 65 hips in the non-overcorrection and overcorrection groups)

**Fig. 4:**
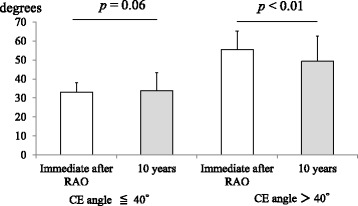
Time course change of center edge (CE) angle and representative radiographs of the patients in the overcorrection group after rotational acetabular osteotomy (RAO). CE angle immediately after RAO significantly decreased with time in the overcorrection group (55.4 ± 9.7 to 49.3 ± 13.2, *p* < 0.01) but not in the non-overcorrection group (33.1 ± 5.0 to 33.8 ± 9.6, *p* = 0.06)

There was no significant difference in the THA-free survival rate between the overcorrection group and the non-overcorrection group in the OA progression survival rate during the 10-year follow-up (Fig. 3). We then compared the time course change in the CE angles in both groups. Interestingly, the CE angle significantly decreased over time in the overcorrection group but not in the non-overcorrection group (Fig. 4). It is likely because the portion of the overhanging in the acetabular roof after RAO was not loaded in daily life that attributed remodeling and resulted in resorption of the overcorrected acetabular roof.

There were no cases of intraoperative and early postoperative complications, such as major neurovascular complications, chisel perforation into the joint, thrombosis of the deep vein, postoperative movement of a rotated acetabulum, and delayed union of the osteotomy site. Further, there was no significant relationship between smoking and the result of RAO.

There was no significant difference in the THA-free survival rate (*p* = 0.10) nor in the non-OA progression rate (*p* = 0.15) among the patients treated with RAO alone, RAO with valgus osteotomy, and RAO with varus osteotomy.

**Fig. 5:**
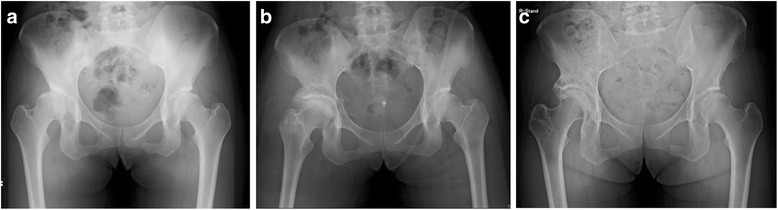
Radiographic image of a 24-year-old woman treated with rotational acetabular osteotomy on her right hip. Plane radiography of the hip showed an AHD, however, the joint space was persisting (**a**). The coverage of femoral head was improved after RAO (**b**). **c** is the radiograph at 12 years after RAO, she did not have any symptoms on her right hip and there was no progression of joint space narrowing

**Fig. 6:**
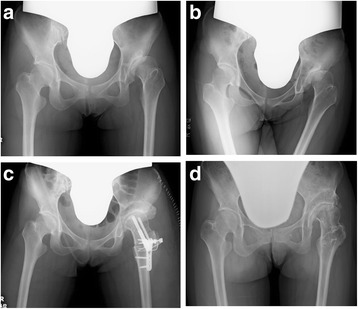
Radiographic image of a 25-year-old woman treated with rotational acetabular osteotomy and valgus intertrochanteric osteotomy on her left hip. The preoperative radiographic image shows AHD with a deformity of the femoral head (**a**). The joint space becomes wider when the hip is adducted, and the position of the loading area of the femoral head becomes horizontal (**b**); thus, we performed RAO with valgus femoral osteotomy (**c**). There is no progression of OA 10 years after the surgery compared with the finding immediately after surgery, and she had no pain or any complaints on her left hip (**d**)

Figure 5a shows the radiographic image of a 24-year-old woman who presented to our department with a history of coxalgia on her right hip, which gradually deteriorated. The plane radiographic image of the hip showed AHD; however, the joint cartilage was preserved. Figure 5b shows the radiographic image immediately after RAO in which the coverage of the femoral head improved. Figure 5c shows the radiographic image 12 years after RAO; she did not have any symptoms on her left hip, and there was no joint space narrowing. Figure 6a shows the plane radiographic image of a 25-year-old woman with AHD and deformity of the femoral head. The joint space became wider when the hip was adducted, and the position of the loading area of the femoral head became horizontal (Fig. 6b); thus, we performed RAO with valgus femoral osteotomy (Fig. 6c). Figure 6d shows the radiographic image 10 years after the surgery; there was no progression of OA compared with the finding immediately after surgery. She had no pain or other complaints on her left hip.

## Discussion

RAO is one of the well-established osteotomies for patients with AHD [4–9, 16, 17]. However, even with this success rate of this surgery, some patients still experience hip pain or progressive OA that may require THA, especially in middle-aged patients [18–25]. Therefore, the primary purpose of the current study was to investigate the risk factors for poor RAO treatment outcomes. For this purpose, we firstly investigated the long-term outcomes of RAO in our institution and found that RAO is a successful treatment for AHD that allows more than 90% of the hips to not required arthroplasty for >10 years. Several authors also reported the long-term follow-up results of RAO. The 10-year survival rate in the current study was lower than that previously reported. It is likely because the mean age at the time of surgery in this study was 36.5 years, which is a little older than that in other reports [11, 26]. Several authors reported that one of the factors predicting poor outcomes was age at the time of surgery [26, 27]. In accordance with these previous reports, age at the time of surgery in the current study is a significant risk factor for THA requirement or OA progression after RAO. As general knowledge, the incidence of articular cartilage degeneration of the hip joint is higher in older patients than in younger patients. Therefore, the outcomes of RAO would be poorer in older patients than in younger patients. Yuasa et al. reported that pre-existing OA was a significant predictor of failure of RAO [28]. In the current study, we classified all hips into 4 stages according to the JOA classification, which is mainly determined using the JSW. However, based on the Cox regression analysis, the JSW itself was not a risk factor of THA or OA progression. In the current study, cases of hips with severe OA (JOA stage 4) were not included; this might be one of the reasons why the JSW did not reveal any significance in the results.

Several authors noted that excessive lateral and anterior correction in the acetabular osteotomy might lead to the cam or pincer type of femoroacetabular impingement (FAI) [6, 12], which may induce residual pain and limit hip motion. Chung et al. reported that in the hips with CE angles over 40°, there might be the risk of FAI, which may lead to OA progression [14]. Siebenrock et al. reported that 29% of the hips in their study had symptomatic impingement after Bernese periacetabular osteotomy (PAO), which is likely due to excessive acetabular roof correction [6]. Myers et al. reported the risk of secondary impingement after PAO [12]. Steppacher et al. also demonstrated a postoperative impingement sign as a predictor of poor outcome following PAO [27]. However, Yasunaga et al. found no significant correlations between a positive crossover sign and radiographic progression of OA, although anterior impingement signs increased after RAO [29]. In the current study, there was no significant difference between the non-overcorrection group and overcorrection group.

Furthermore, we assessed the time course change in the acetabular roof after RAO. This finding indicates that in overcorrected hips, the mechanical stress was not loaded on the edge of the rotated acetabular segment. As the result of the joint stress change, the edge of the acetabular segment might be remodeled.

Several authors reported the harmful influence of smoking on bone healing after fracture [30–34]. However, in the current study, there was no significant influence of smoking on either the result of RAO or union of a rotated acetabulum. This result might be due to the reasons following below; the osteotomy aspect was relatively large and rotated fragment and iliac bone were well fixed by using screws. Furthermore, blood flow of bone marrow of ilium abound which is favorite environment for bone remodeling.

In the current study, there was no significant difference in the survival rate among the patients whose hips were treated with different surgeries. This might be because the number of the hips treated with an additional femoral osteotomy was relatively too small to be used for comparison.

One limitation of this study was that we only addressed anteroposterior radiographs for the evaluation of hip status, despite the three-dimensional structure of the hips. However, in the current study, sufficient coverage was achieved, and the statistical survival analysis showed that there was no harmful influence in the overcorrection group compared to the nonovercorrection group. Another limitation was that most of the THAs were performed in older patients; thus, there might be a tendency that we subliminally avoided THAs in younger patients. This bias might have an influence on the result of the current study; however, the Cox regression analysis was performed not only for THA requirement but also for OA progression, and age at the time of surgery influenced both THA requirement and OA progression. Lastly, the study was a retrospective study with a relatively small number of patients. To determine adequate correction angles for the acetabular roof in RAO, prospective interventions in a larger population will be needed to elucidate accurate evaluations of long-term outcomes in RAO for future studies.

## Conclusion

RAO is an effective surgical procedure for symptomatic patients with AHD that prevents OA progression and protects the hips from undergoing THA. In the current study, 23.3% of the hips showed OA progression, and THA was required in 7.7% of the hips after RAO during the 10-year follow-up. However, older patients have a higher risk for both OA progression and THA requirement. In RAO, acetabular correction should be sufficient to obtain an efficient femoral head coverage.

## Abbreviations

AC angle: Acetabular roof obliquity
AHD: Adulthood hip dysplasia
CE angel: Center edge angle
FAI: Femoroacetabular impingement
JOA: Japanese Orthopedic Association
JSW: Joint space width
OA: Osteoarthritis
PAO: Periacetabular osteotomy
RAO: Rotational acetabular osteotomy
ROC curve: Receiver operating characteristic curve
THA: Total hip arthroplasty
